# Synthesis, Characterization, and In Vitro Cytotoxic Evaluation of Neodymium-Doped Cobalt Ferrite Nanoparticles on Human Cancer Cell Lines

**DOI:** 10.3390/ma18163911

**Published:** 2025-08-21

**Authors:** Slaviţa Rotunjanu, Armand Gogulescu, Narcisa Laura Marangoci, Andrei-Ioan Dascălu, Marius Mioc, Roxana Racoviceanu, Alexandra Mioc, Tamara Maksimović, Oana Eșanu, Gabriela Antal, Codruţa Șoica

**Affiliations:** 1 Faculty of Pharmacy, “Victor Babes” University of Medicine and Pharmacy, 300041 Timisoara, Romania; slavita.rotunjanu@umft.ro (S.R.); marius.mioc@umft.ro (M.M.); babuta.roxana@umft.ro (R.R.); alexandra.mioc@umft.ro (A.M.); tamara.maksimovic@umft.ro (T.M.); oana.esanu@umft.ro (O.E.); gabriela.antal@umft.ro (G.A.); codrutasoica@umft.ro (C.Ș.); 2 Research Center for Experimental Pharmacology and Drug Design (X-Pharm Design), “Victor Babes” University of Medicine and Pharmacy, 300041 Timisoara, Romania; 3 Faculty of Medicine, “Victor Babes” University of Medicine and Pharmacy, 300041 Timisoara, Romania; 4 Institute of Macromolecular Chemistry ‘Petru Poni’, 700487 Iasi, Romania; nmarangoci@icmpp.ro (N.L.M.); idascalu@icmpp.ro (A.-I.D.)

**Keywords:** neodymium-doped magnetic nanoparticles, cobalt ferrites, cytotoxicity, cell viability, anticancer

## Abstract

Cancer is still the world’s most prevalent cause of death, and the limited efficacy of current treatments highlights the requirement for new therapeutic approaches. In this study, neodymium (Nd)-doped cobalt ferrite (CoFe_2₋z_Nd_z_O_4_, z = 0; 0.01; 0.02; 0.03; 0.05; 0.1) nanoparticles (Nd0-Nd5) were synthesized via the combustion method. The structural, morphological, and magnetic properties were characterized using X-ray diffraction (XRD), Fourier-transform infrared spectroscopy (FTIR), vibrating sample magnetometry (VSM), and scanning transmission electron microscopy (STEM) analysis. The synthesized compounds demonstrated single-phase spinel structures, with morphological differences observed between undoped and Nd-doped samples. The biological activity of the nanoparticles was evaluated on immortalized human keratinocytes (HaCaT) and on cancer cell lines: melanoma (A375), breast adenocarcinoma (MCF-7), and pancreatic carcinoma (PANC-1). The cytotoxic effects of Nd0-Nd5 (50–1000 μg∙mL^−1^) were assessed through Alamar Blue and lactate dehydrogenase (LDH) release assays. The results indicated a dose-dependent cytotoxic effect in cancer cell lines. Changes in cell morphology, suggesting the induction of the apoptotic processes, were observed through immunofluorescence staining of F-actin and nuclei. These findings highlight the potential of Nd-doped cobalt ferrite nanoparticles as selective anticancer agents, warranting further investigation to fully elucidate their mechanisms of action and therapeutic applicability.

## 1. Introduction

Malignant pathologies stand today as the main leading cause of death worldwide; despite huge improvements in anticancer therapy, modern medicine still lacks the tools to avoid drug resistance or the severe side effects that accompany current treatments [1,2]. Therefore, the search for new therapeutic alternatives continues, including materials of various origins. Nanotechnology has established itself in the biomedical field as a powerful weapon against various pathologies, including cancer, where it provides both therapeutic and diagnostic agents, sometimes combined in multifunctional platforms [3,4].

Among the multitude of nanoparticles synthesized to date, metallic nanoparticles carry particular importance due to their tunable properties, flexible surface functionalization, and straightforward synthetic approach. Metallic nanoparticles can be obtained in a variety of size range with low dispersity indexes, are biocompatible and inert, are able to penetrate the target organs by crossing biological barriers and, more importantly, may induce an enhanced biological activity due to their specific particle size (<100 nm) and high surface area that enable their binding to cellular compounds such as proteins and nucleic acids [5].

Magnetic nanoparticles have gained interest in the biomedical area due to their ability to be manipulated through an external magnetic field to generate heat, which increases tumor temperature above the normal body temperature, thus leading to its elimination (hyperthermia); this effect can be combined with drug delivery within multipurpose platforms with high anticancer efficiency [6]. However, the intrinsic cytotoxic activity of such nanoparticles was also explored and revealed that cancer cells are more sensitive to metallic nanoparticles than normal cells. Mechanisms suggested for such effects include the generation of reactive oxygen species (ROS), the activation of caspase 3, the increase in mitochondrial permeability, and the fragmentation of DNA molecules, overall activating various signaling pathways that lead to cell death [7]. Numerous evidences suggest that nanoparticles, particularly iron oxide-based, are able to interact with the host immune system, thus stimulating the immune recognition of the tumor through yet unknown mechanisms [8]. Iron oxide nanoparticles can be doped with other elements that occupy various positions in the crystal lattice and induce the alteration of their resulting physicochemical, biological, electrical, and optical properties [9]. A challenge in obtaining doped iron oxide nanoparticles useful for the biomedical field is to find an element with strong magnetic properties combined with stability and biocompatibility. A strategy was to replace divalent iron ions with cobalt ions, which are more anisotropic and display comparable ionic size [10]. Cobalt-doped iron oxide nanoparticles, also known as cobalt ferrites, possess high intrinsic magneto-crystalline anisotropy values, which trigger significantly higher coercivity compared to pure iron oxide nanoparticles. Unlike other ferrites, cobalt ferrite exhibits an inverse spinel structure with divalent ions occupying octahedral sites in the crystal lattice, while trivalent ions are distributed evenly between tetrahedral and octahedral sites [11]. Cobalt is an interesting choice as a doping element given its intrinsic cytotoxic properties; cobalt nanoparticles were tested against breast and colon cancer cells, where they easily penetrated cell membranes and induced cell apoptosis and death in a significant proportion while inducing low cytotoxic effects in healthy cells even at high concentrations [12].

The doping process may go even further by employing rare earth elements as supporting material in an effort to increase anticancer efficiency. Various rare earth metals were assessed, among which Nd stands as one of the most widely studied in terms of bio-imaging applications due to its excitation and emission lines within the first NIR biological transparency window [13]. Nd also displays intrinsic anticancer properties by binding to double-stranded DNA with high equilibrium association constants. Additionally, Nd in high concentration is able to induce the strong condensation of the DNA’s double helix, thus promoting its collapse in a similar manner to conventional chemotherapy agents [14]. However, studies on Nd-doped cobalt ferrites are still scarce, particularly in terms of the molecular underlying mechanisms.

We aimed to develop Nd-doped cobalt ferrites by means of combustion, a straightforward synthetic approach. The doped ferrites were then assessed in terms of physicochemical properties by employing X-ray diffraction (XRD), Fourier-transform infrared spectroscopy (FTIR), vibrating sample magnetometer (VSM), and scanning transmission electron microscopy (STEM) analysis. Their biological assessment was carried out using Alamar Blue and LDH release assays on normal HaCaT cell line and on cancer cell lines (A375, MCF-7, and PANC-1), complemented by immunofluorescence staining to evaluate the morphological changes that were associated with the cytotoxic effects.

## 2. Materials and Methods

### 2.1. Chemicals

For the synthesis, the following raw materials were used: iron nitrate Fe(NO_3_)_3_·9H_2_O, cobalt nitrate Co(NO_3_)_2_·6H_2_O, Nd chloride NdCl_3_·6H_2_O, and glycine C_2_H_5_NO_2_. All reagents were analytical grade and were purchased from Sigma-Aldrich (Darmstadt, Germany).

### 2.2. Synthesis by Combustion Method

A combustion method using cobalt nitrate (0.02 moles), iron nitrate (0.04 moles), and glycine (0.09 moles) as the primary precursors was employed to obtain 0.02 moles of cobalt ferrite. The metal nitrates served as oxidizing agents, while glycine functioned as the fuel. The mixture was heated in a Berzelius glass at 60 °C until the color of the solution changed to brown. Meanwhile, a porcelain crucible was preheated in a heating mantle at approximately 350 °C; the temperature was maintained for the entire reaction period. Once the precursor solution developed a viscous consistency due to solvent evaporation, it was transferred into the preheated crucible. As the viscosity further increased, the mass self-ignited, initiating a self-sustained combustion reaction. During this reaction, the appearance of yellow flames indicated that a high temperature was reached. The combustion, which lasted around 10 s, resulted in a black, porous, and brittle powder.

The synthesis of Nd-doped cobalt ferrite followed the same steps, with the addition of Nd chloride as the dopant precursor. In these formulations, the amount of glycine and cobalt nitrate was the same as in the previous method, while the iron nitrate and Nd chloride proportions changed according to Table 1. During the reaction, white gases were released, and the combustion duration was 4 s longer than the undoped counterpart. Additionally, the final powders of the Nd-doped samples appeared bulkier and exhibited a noticeably spongier texture compared to the undoped cobalt ferrite.

### 2.3. Characterization Methods

XRD analysis was conducted with a diffractometer (Rigaku SmartLab, Tokyo, Japan) that operates at 45 kV and 200 mA and is equipped with a Cu anode (1.5406 Å wavelength) [15]. Phase identification was carried out using the Crystallography Open Database, and Rietveld refinement of the diffraction patterns was conducted with a dedicated analysis package: Rigaku SmartLab Studio II software. FTIR was conducted using the Shimadzu IR Affinity-1S spectrophotometer (Shimadzu Scientific Instruments Inc., Columbia, MD, USA), with a range of 400–4000 cm^−1^, resolution of 4 cm^−1^, and 40 scans/sample. Sample preparation was carried out using the standard KBr pellet technique. The characterization of the magnetic properties was carried out with VSM-LakeShore 8607 (Shore Cryotronics, Westerville, OH, USA). Prior to all measurements, all samples were demagnetized in an alternating magnetic field. Magnetization curves were measured at room temperature under an applied field ranging from +30 to −30 KOe. Morphological evaluation was performed using the Hitachi High-Tech HT7700 (Hitachi High-Technologies Corporation, Tokyo, Japan) at 120 kV in high contrast mode. This instrument was equipped with an EDX detector for elemental analysis, with SAED apertures, and with a STEM module. The sample preparation from their water suspension was conducted by drop casting on 300 mesh carbon-coated copper grids obtained from Ted Pella Inc. (Redding, CA, USA), followed by 24 h vacuum-drying at 25 °C.

### 2.4. Cell Culture

In this study, we used immortalized human keratinocytes (HaCaT) cells, purchased from CLS Cell Lines Service GmbH (Eppelheim, Germany), as well as human melanoma (A375), human breast adenocarcinoma (MCF-7), and human pancreatic carcinoma (PANC-1) cells, purchased from American Type Culture Collection (ATCC, Lomianki, Poland). HaCaT, A375, and PANC-1 cells were grown in Dulbecco’s Modified Eagle Medium (DMEM), whereas MCF-7 cells were grown in Eagle’s Minimum Essential Medium (EMEM). All media were supplemented with 10% fetal bovine serum (FBS) and 1% antibiotics (penicillin–streptomycin 100 IU·mL^−1^), presenting normal proliferation in culture. Cells were grown in 5% CO_2_ atmosphere at 37 °C.

### 2.5. Cell Viability Assessment

The viability of all cell lines (HaCaT, A375, MCF-7, and PANC-1) was evaluated after 48 h treatment with the newly obtained compounds Nd0-Nd5 (50, 100, 250, 500, and 1000 μg·mL^−1^), using the Alamar Blue assay. The cells were grown in 96-well culture plates (1 × 10^4^ cells/well) and incubated at 37 °C and 5% CO_2_. After reaching an appropriate confluence (80–90%), the old media were removed and the cells were stimulated with Nd0-Nd5 for 48 h. The cells were then stained with Alamar Blue 0.01% solution and incubated for another 3 h; absorbance was measured at 570/600 nm using a multimode microplate reader (BioTek Synergy HTX, Agilent Technologies, Santa Clara, CA, USA).

### 2.6. Lactate Dehydrogenase (LDH) Assay

The cytotoxic potential of Nd0-Nd5 (50, 100, 250, 500, and 1000 μg·mL^−1^) on A375, MCF-7, and PANC-1 cancer cells was determined by measuring the extracellular release of the cytosolic LDH, according to a previously established protocol [16]. Briefly, the cells were grown in 96-well plates (1 × 10^4^ cells/well), incubated until 80–90% confluence, and treated with the newly obtained compounds (Nd0-Nd5). After 48 h, 50 μL of culture medium was collected from each well and transferred, together with 50 µL of LDH reaction mixture, to a new 96-well plate. After 30 min incubation at room temperature, the reaction was stopped using 50 µL of stop solution. The absorbance was measured at 490/680 nm (xMark™ Microplate Spectrophotometer, Bio-Rad, Hercules, CA, USA).

### 2.7. Immunofluorescence Assay

A375, MCF-7, and PANC-1 cells were seeded in 6-well plates until reaching an appropriate confluency and treated for 48 h with Nd0, Nd1, Nd2, Nd3, Nd4, and Nd5 at two representative concentrations (500 and 1000 μg·mL^−1^). According to a previously established protocol [17], cells were washed (3 times with cold PBS), fixed (4% paraformaldehyde), permeabilized (0.1% Triton X in PBS for 15 min), blocked (30% FCS), and washed again (3 times with cold PBS). Actin filaments (F-actin) were labeled using Alexa Fluor™ 594 Phalloidin (Thermo Fisher Scientific, Boston, MA, USA)—30 min incubation at room temperature. The nuclei were stained and morphologically analyzed using the Hoechst 34580 staining (Thermo Fisher Scientific, Boston, MA, USA)—5 min incubation at room temperature. The morphological assessment was analyzed using the EVOS™ M5000 Imaging System (Thermo Fisher Scientific, Boston, MA, USA).

### 2.8. Statistical Analysis

The statistical difference between treatment and control was determined using the one-way ANOVA analysis and the Dunnett’s multiple comparisons post-test (GraphPad Prism version 10.5.0, GraphPad Software, San Diego, CA, USA), respectively (* *p* < 0.05, ** *p* < 0.01, and *** *p* < 0.001).

## 3. Results

### 3.1. X-Ray Diffraction

**Figure 1:**
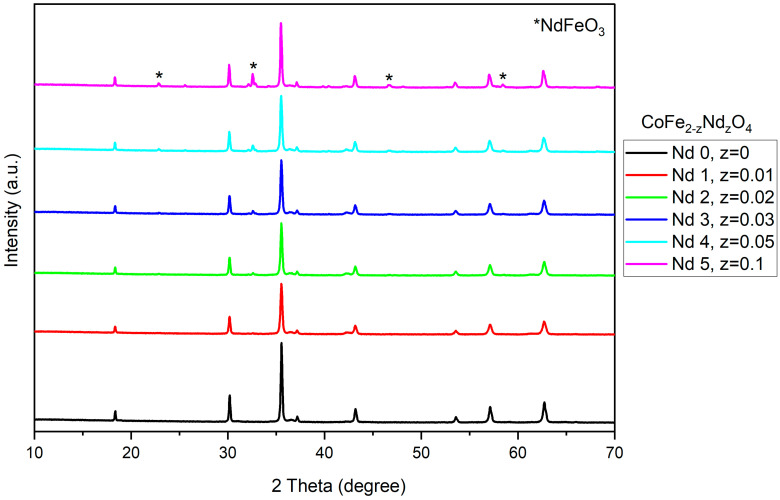
X-ray diffraction spectra of cobalt ferrite (Nd0) and Nd-doped cobalt ferrite (Nd1-Nd5). Samples present the characteristic diffraction pattern for cobalt ferrite. Nd3-Nd5 show secondary phase NdFeO_3_.

As revealed by the allure of the peaks, all samples exhibit a high degree of crystallinity. In sample Nd0, the spectrum contains bands that show a single-phase cubic cobalt ferrite Fd3m structure (Figure 1). Samples Nd 1 and Nd 2 exhibit no additional bands, thus indicating the successful incorporation of Nd in the ferrite structure. Phase identification was achieved with the COD card 96-153-3164. The diffractions peaks found at angles 18.22°, 30.10°, 35.48°, 37.11°, 43.13°, 53.50°, 57.05°, and 62.66° were attributed to the crystallographic planes (111), (220), (311), (222), (400), (422), (511), and (440), thus confirming the formation of cobalt ferrite. In sample Nd3, only a small peak located at 32.54° was recorded, indicating that the secondary phase occurred in trace amounts. In samples Nd4 and Nd5, supplementary diffraction peaks were noted, indicating the formation of a secondary phase that was identified as orthorhombic perovskite NdFeO_3_ using the COD card 96-200-3125. The peaks present at angles 2θ 22.79°, 32.54°, 46.63°, and 58.40° were assigned to the crystallographic planes (110), (112), (220), and (204). One can notice that with the increase in Nd content, the NdFeO_3_ peaks became more intense, particularly the one located at 32.54°.

Table 2 displays the α lattice parameter calculated for every sample by means of Rietveld refinement. One can notice that when Nd was added as a doping agent, a small increase in the lattice parameter was recorded, which confirms Nd inclusion in the spinel structure. Moreover, as the Nd content progressively increased, the lattice parameter increased.

### 3.2. Fourier-Transform Infrared Spectroscopy

**Figure 2:**
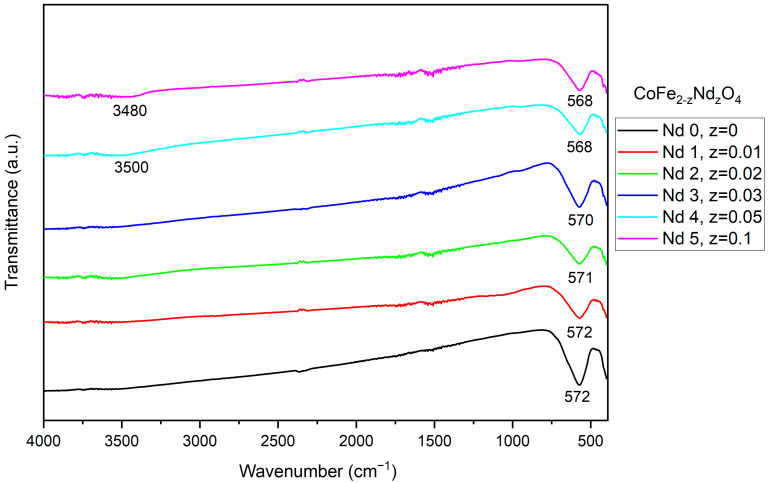
Fourier-transform infrared spectra of cobalt ferrite (Nd0) and Nd-doped cobalt ferrite (Nd1–Nd5). Samples exhibit the absorption bands typical of cobalt ferrite. Nd doping induced a slight shift towards lower wavenumbers.

The FTIR spectra (Figure 2) showed that all samples have an intense absorption band accompanied by another, partially visible, band. Both bands are located between 400 and 800 cm^−1,^ which is characteristic of the metal–oxygen stretching vibration in cobalt ferrite. In addition, a small shift in the band can be noticed as the Nd content increases. Samples Nd4 and Nd5 show a wide and small band around 3500 cm^−1^, characteristic of -OH stretching vibration.

### 3.3. Vibrational Sample Magnetometry

**Figure 3:**
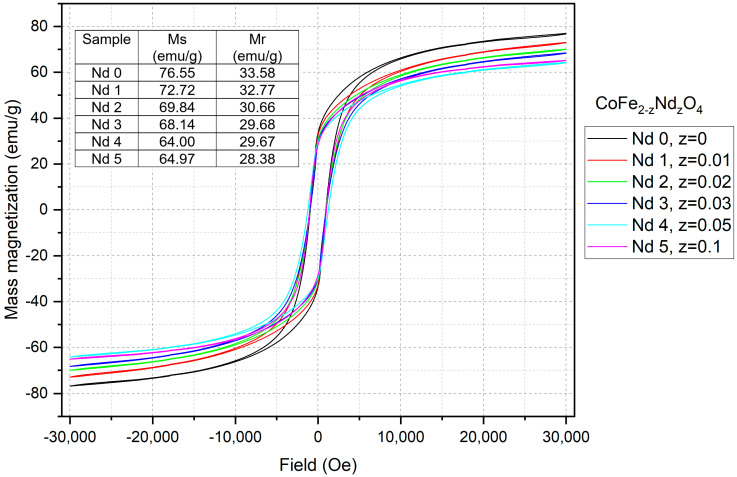
Magnetic hysteresis loops of cobalt ferrite (Nd0) and Nd-doped cobalt ferrite (Nd1–Nd5) recorded at room temperature. Samples display ferromagnetic behavior. Ms and Mr values decrease as Nd content increases.

The recorded sample magnetic properties are presented in Figure 3. A similar behavior can be noticed in all samples, with the hysteresis maintaining the same shape while the intensity varies. The mass magnetization (Ms) recorded for Nd0 (cobalt ferrite) is 76.55 emu∙g^−1^, while the remnant magnetization (Mr) has a value of 33.58 emu∙g^−1^. For the Nd-doped samples Nd1–Nd5, a decrease in both Ms and Mr can be noticed with the increase in the Nd content; as an example, in sample Nd5, the Ms decreased to 64.97 emu∙g^−1^ and Ms to 28.38 emu∙g^−1^.

### 3.4. Scanning Transmission Electron Microscopy

**Figure 4:**
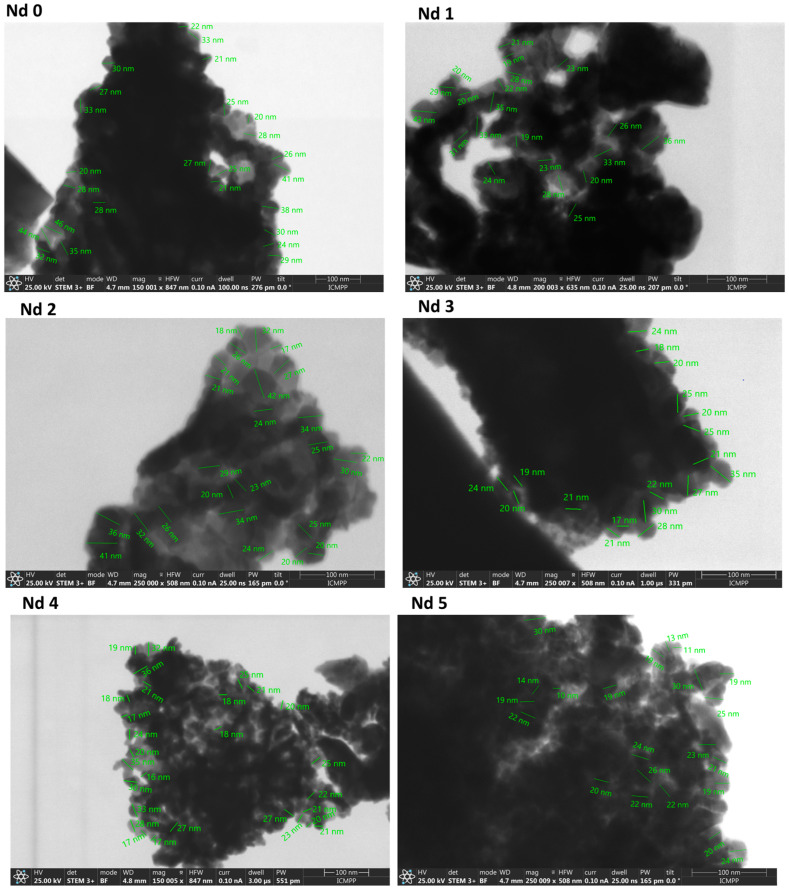
Scanning transmission electron microscopy images of cobalt ferrite (Nd0) and Nd-doped cobalt ferrite (Nd1–Nd5). Samples show agglomerated nanoparticles forming clusters. Nd doping led to a decrease in particle size.

All samples exhibited a pronounced tendency towards agglomeration and stacking, leading to cluster formation, which made it difficult to evaluate the particle size. The STEM images show the formation of both doped and undoped cobalt ferrite nanoparticles (Figure 4). For the undoped cobalt ferrite Nd0, the particle size ranges between 20 and 46 nm, while for the doped samples, a modest reduction was noted with diameters varying between 19 and 43 nm in Nd1 and between 17 and 42 nm in Nd2. Samples Nd3 and Nd4 have comparable dimensions of 17–35 nm and 16–36 nm, respectively. In sample Nd5, the size decrease is more obvious compared to the undoped sample, with diameters ranging between 10 and 30 nm. Thus, one may state that the inclusion of Nd into the ferrite lattice resulted in a decrease in particle dimension in direct correlation with the Nd content.

### 3.5. Energy Dispersive Analysis by X-Ray

**Figure 5:**
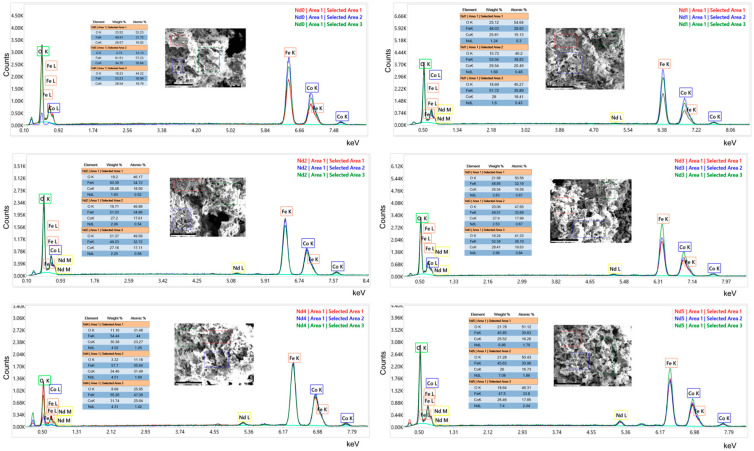
Energy-dispersive X-ray spectroscopy spectra of cobalt ferrite (Nd0) and Nd-doped cobalt ferrite nanoparticles (Nd1–Nd5). Co and Fe peaks confirm the cobalt ferrite composition in all samples. Nd-doped samples show additional Nd typical peaks.

The EDAX analysis presented in Figure 5 confirms that cobalt ferrite was formed, as well as the presence of Nd in the doped samples (Nd1–Nd5).

In sample Nd 0, the EDAX analysis reveals the specific peaks of cobalt ferrite with the Kα line for Fe located at approximately 6.4 keV and the one for Co around 6.9 keV. The peak located at 7.0 keV was assigned to FeKβ, which overlaps CoKα. A smaller peak can be noticed around 7.6 keV and can be attributed to CoKβ. The 0–1 keV region hosts Lα lines for iron and cobalt and the Kα line for oxygen. The content of oxygen was not rigorously obtained due to the limitation of this technique to quantify light elements. For the doped samples, the Nd lines are present, marked by a distinct peak located around 5.2 keV and assigned to NdLα; an M line around 0.8 keV overlapped in the 0–1 keV domain.

### 3.6. Nd Compounds Decrease Cancer Cell Viability

**Figure 6:**
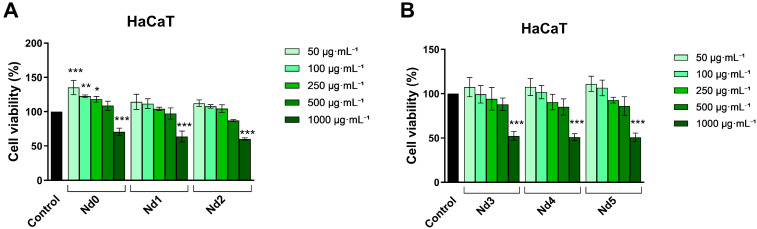
The viability of normal HaCaT cells treated for 48 h with Nd0, Nd1, and Nd2 (**A**) and with Nd3, Nd4, and Nd5 (**B**) at 50, 100, 250, 500, and 1000 μg·mL^−1^. These results are presented as cell viability % normalized to control (100%, cells without treatment) and represent mean values ± SD of three independent experiments performed in triplicate. (* *p* < 0.05, ** *p* < 0.01 and *** *p* < 0.001).

**Figure 7:**
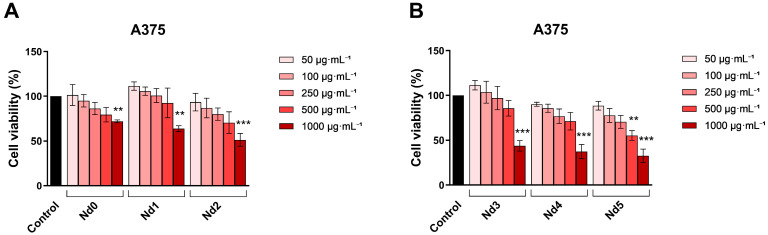
The viability of A375 cancer cells treated for 48 h with Nd0, Nd1, and Nd2 (**A**) and with Nd3, Nd4, and Nd5 (**B**) at 50, 100, 250, 500, and 1000 μg·mL^−1^. These results are presented as cell viability % normalized to control (100%, cells without treatment) and represent mean values ± SD of three independent experiments performed in triplicate. (** *p* < 0.01 and *** *p* < 0.001).

**Figure 8:**
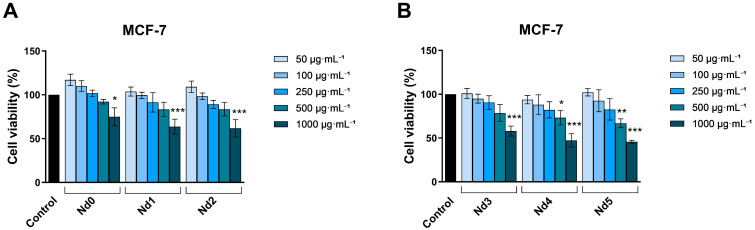
The viability of MCF-7 cancer cells treated for 48 h with Nd0, Nd1, and Nd2 (**A**) and with Nd3, Nd4, and Nd5 (**B**) at 50, 100, 250, 500, and 1000 μg·mL^−1^. These results are presented as cell viability % normalized to control (100%, cells without treatment) and represent mean values ± SD of three independent experiments performed in triplicate. (* *p* < 0.05, ** *p* < 0.01 and *** *p* < 0.001).

**Figure 9:**
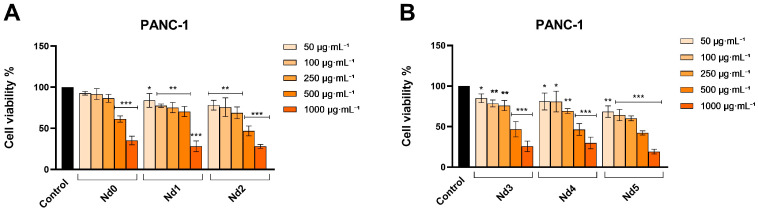
The viability of PANC-1 cancer cells treated for 48 h with Nd0, Nd1, and Nd2 (**A**) and with Nd3, Nd4, and Nd5 (**B**) at 50, 100, 250, 500, and 1000 μg·mL^−1^. These results are presented as cell viability % normalized to control (100%, cells without treatment) and represent mean values ± SD of three independent experiments performed in triplicate. (* *p* < 0.05, ** *p* < 0.01 and *** *p* < 0.001).

The potential antitumor properties of Nd complexes (Nd0, Nd1, Nd2, Nd3, Nd4, and Nd5) in normal HaCaT cells and in A375, MCF-7, and PANC-1 cancer cells were investigated following a 48 h treatment with increasing concentrations—50, 100, 250, 500, and 1000 µg·mL^−1^, respectively. When tested, Nd-doped compounds affected cell viability in a dose-dependent manner while expressing different levels of cytotoxicity depending on the cell type (Figure 6, Figure 7, Figure 8 and Figure 9). A common observation for all six studied compounds is that lower concentrations (50 µg·mL^−1^–500 µg·mL^−1^) did not exert any cytotoxic effects on HaCaT cells, in some cases even increasing the number of viable cells, with the viability values ranging from 135.27 ± 10.48% vs. control after 50 µg·mL^−1^ Nd0 treatment, to 85.03 ± 9.12% after 500 µg·mL^−1^ Nd4 treatment. On the contrary, the highest concentration (1000 µg·mL^−1^) reduced the viability of HaCaT cells, to 70.60 ± 5.35% (Nd0), 63.83 ± 7.88% (Nd1), 60.26 ± 1.07% (Nd2), 52.34 ± 4.81% (Nd3), 50.89 ± 3.96% (Nd4), and 50.76 ± 4.85% (Nd5) (Figure 6).

Against the A375 cell line, the tested compound exerted a strong cytotoxic activity (Figure 2); the viability of melanoma cells was most affected at the concentration of 1000 µg·mL^−1^, by Nd5 (32.67 ± 7.51%), followed by Nd4 (37.43 ± 7.85%), Nd3 (43.84 ± 5.88), Nd2 (51.28 ± 7.06%), Nd1 (63.65 ± 3.33%), and finally Nd0 (71.88 ± 1.57%) (Figure 7).

A similar dose-dependent trend was observed in MCF-7 line, where there was a significant drop in cell viability after stimulation with 1000 µg·mL^−1^ concentration of Nd5 (45.79 ± 2.19%), Nd4 (47.34 ± 7.55%), Nd3 (57.90 ± 5.67%), Nd2 (61.78 ± 9.78%), Nd1 (63.65 ± 8.44%), and Nd0 (74.87 ± 10.36%), and 500 µg·mL^−1^ concentration of Nd5 (66.75 ± 5.05%) and Nd4 (73.18 ± 8.26%) (Figure 8).

In PANC-1 cancer cells, all of the applied treatments caused a gradual and dose-dependent decline of cell viability (Figure 9). However, the cytotoxic potential was highly dependent on the tested complex. Thus, a statistically significant reduction in the percentage of viable cells was obtained at all concentrations for Nd1-Nd5, while Nd0 produced a significant effect only starting with the concentrations of 250 µg·mL^−1^ (Figure 9). Specifically, the highest concentrations tested (500 and 1000 µg·mL^−1^) of Nd0-Nd5 reduced cell viability of PANC-1 cells as follows: 61.28 ± 3.81% and 35.16 ± 5.21% (Nd0),70.36 ± 6.19% and 28.10 ± 6.53% (Nd1), 46.85 ± 5.97% and 28.03 ± 2.32% (Nd2), 46.75 ± 9.38% and 25.67 ± 6.53% (Nd3), 46.50 ± 7.24% and 29.84 ± 7.20% (Nd4) and to 42.24 ±2.52% and 19.21 ± 2.83% (Nd5) (Figure 4).

The calculated IC_50_ values of the tested compounds in HaCaT, A375, MCF-7, and PANC-1 cells (Table 3) indicate a similar anti-tumor effect against the cancerous cell lines and a lack of cytotoxic effect against the normal HaCaT cell line.

### 3.7. Nd Compounds Increase Lactate Dehydrogenase Release

**Figure 10:**
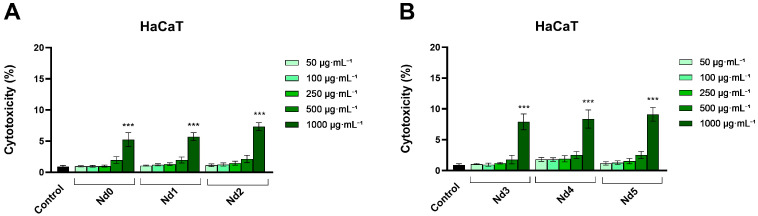
Lactate dehydrogenase (LDH) release percentages in HaCaT cancer cells after 48 h treatment with Nd0, Nd1, and Nd2 (**A**) and after treatment with Nd3, Nd4, and Nd5 (**B**) at 50, 100, 250, 500, and 1000 μg·mL^−1^. These results are presented as LDH Release (%) and represent mean values ± SD of three independent experiments performed in triplicate. (*** *p* < 0.001).

**Figure 11:**
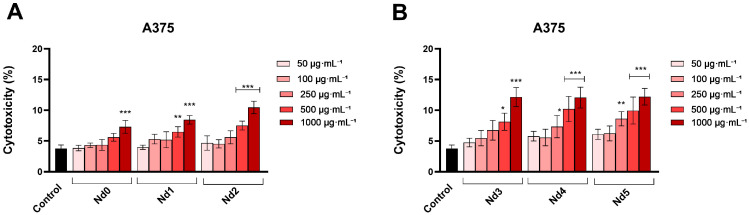
Lactate dehydrogenase (LDH) release percentages in A375 cancer cells after 48 h treatment with Nd0, Nd1, and Nd2 (**A**) and after treatment with Nd3, Nd4, and Nd5 (**B**) at 50, 100, 250, 500, and 1000 μg·mL^−1^. These results are presented as LDH Release (%) and represent mean values ± SD of three independent experiments performed in triplicate. (* *p* < 0.05, ** *p* < 0.001 and *** *p* < 0.001).

**Figure 12:**
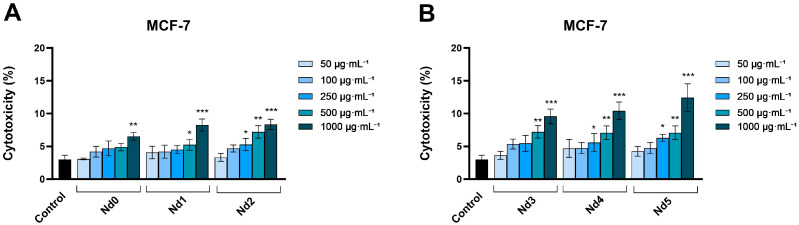
Lactate dehydrogenase (LDH) release percentages in MCF-7 cancer cells after 48 h treatment with Nd0, Nd1, and Nd2 (**A**) and after treatment with Nd3, Nd4, and Nd5 (**B**) at 50, 100, 250, 500, and 1000 μg·mL^−1^. These results are presented as LDH Release (%) and represent mean values ± SD of three independent experiments performed in triplicate. (* *p* < 0.05, ** *p* < 0.001 and *** *p* < 0.001).

**Figure 13:**
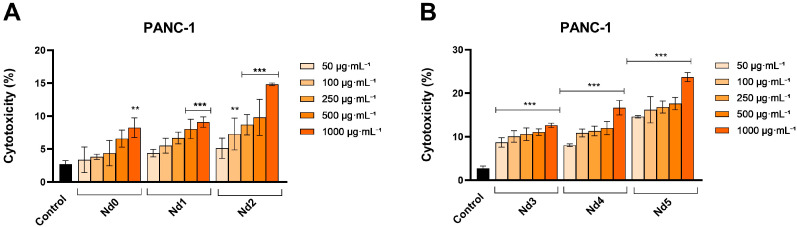
Lactate dehydrogenase (LDH) release percentages in PANC-1 cancer cells after 48 h treatment with Nd0, Nd1, and Nd2 (**A**) and after treatment with Nd3, Nd4, and Nd5 (**B**) at 50, 100, 250, 500, and 1000 μg·mL^−1^. These results are presented as LDH Release (%) and represent mean values ± SD of three independent experiments performed in triplicate. ( ** *p* < 0.001 and *** *p* < 0.001).

The cytotoxic potential of Nd compounds (50, 100, 250, 500, and 1000 µg·mL^−1^) was assessed in HaCaT, A375, MCF-7, and PANC-1 cells by quantifying the lactate dehydrogenase (LDH) released in culture medium after cell membrane rupture or alteration at 48 h post-treatment (Figure 10, Figure 11, Figure 12 and Figure 13).

In HaCaT cells, the tested compounds exhibited cytotoxic potential only at the highest concentration (1000 µg·mL^−1^) (Figure 10). These results are consistent with the ones obtained in the cell viability assay and show that, at lower concentrations (<500 µg·mL^−1^), the compounds did not induce a significant cytotoxic effect in normal cells, suggesting a degree of selectivity toward cancer cells.

In A375 cells, only the highest concentration of Nd0 (1000 μg·mL^−1^) induced a cytotoxic effect vs. control (7.31 ± 1.04% vs. 3.77 ± 0.59%) (Figure 11). Nd1-Nd5 significantly increased the LDH release vs. control (3.77 ± 0.59%) when tested at both 500 μg·mL^−1^ and 1000 μg·mL^−1^, respectively. The obtained values were as follows: 6.48 ± 0.84%% and 8.45 ± 0.69% (Nd1), 7.52 ± 0.72% and 10.45 ± 1.01% (Nd2), 8.14 ± 1.39% and 12.15 ± 1.54 (Nd3), 10.22 ± 2.04% and 12.07 ± 1.69% (Nd4) and 9.96 ± 2.18 and 13.14 ± 1.35% (Nd5).

Treatment of MCF-7 cells with Nd0-Nd5 (50, 100, 250, 500, and 1000 µg·mL^−1^) induced a similar cytotoxic effect, observed by the significant increase in LDH release when the cells were treated with the highest concentrations of the compounds. The treatment with 1000 μg·mL^−1^ of Nd0-Nd5 increased LDH release vs. control (3.03 ± 0.64%), as follows: 6.53 ± 0.57% (Nd0), 8.25 ± 0.91% (Nd1), 8.37 ± 0.79% (Nd2), 9.57 ± 1.22% (Nd3), 10.43 ± 1.72% (Nd4) and 12.76 ± 2.11% (Nd5) (Figure 12).

In PANC-1 cells, the results show that Nd compounds caused a concentration-dependent leakage of LDH, starting with the lowest concentration of 50 μg·mL^−1^ to the highest concentration of 1000 μg·mL^−1^ (Figure 13). Specifically, Nd0 increased LDH release percentage from 2.72 ± 0.53% (control) to 8.28 ± 1.48% (1000 μg·mL^−1^). An increase in cytotoxicity was also obtained for Nd1: 8.04 ± 1.47% (500 μg·mL^−1^), 9.09 ± 0.77% (1000 μg·mL^−1^), and for Nd2: 9.80 ± 2.73% (500 μg·mL^−1^), 14.82 ± 0.20 (1000 μg·mL^−1^). Compounds Nd3-Nd5 produced a dose-dependent cytotoxic effect starting with the lowest concentration tested (50 μg·mL^−1^) up to 1000 μg·mL^−1^. Specifically, Nd3 increased LDH release vs. control (2.72 ± 0.53%), as follows: 8.70 ± 1.07% (50 μg·mL^−1^), 10.07 ± 1.31% (100 μg·mL^−1^), 10.58 ± 1.42% (250 μg·mL^−1^), 11.06 ± 0.74% (500 μg·mL^−1^) and 12.64 ± 0.50% (1000 μg·mL^−1^). Nd4 increased LDH release up to 8.06 ± 0.30% (50 μg·mL^−1^), 10.87 ± 0.90% (100 μg·mL^−1^), 11.35 ± 1.01% (250 μg·mL^−1^), 11.99 ± 1.51% (500 μg·mL^−1^) and 16.67 ± 1.66% (1000 μg·mL^−1^), while Nd5 exhibited the highest increase in LDH release, as follows: 14.61 ± 0.26% (50 μg·mL^−1^), 16.19 ± 3.03% (100 μg·mL^−1^), 16.83 ± 1.37% (250 μg·mL^−1^), 17.64 ± 1.41% (500 μg·mL^−1^) and 23.72 ± 1.03% (1000 μg·mL^−1^).

### 3.8. Nd Compounds Induce Cell Morphology Changes

**Figure 14:**
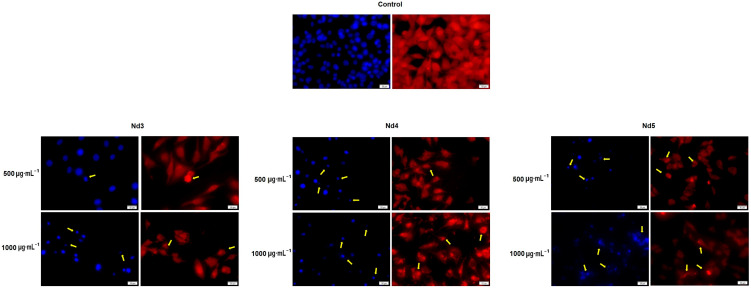
Aspect of the nuclei and F-actin fibers in A375 cells treated for 48 h with Nd3, Nd4, and Nd5 at two representative concentrations (500 and 1000 μg·mL^−1^). The scale bars indicate 50 µm.

**Figure 15:**
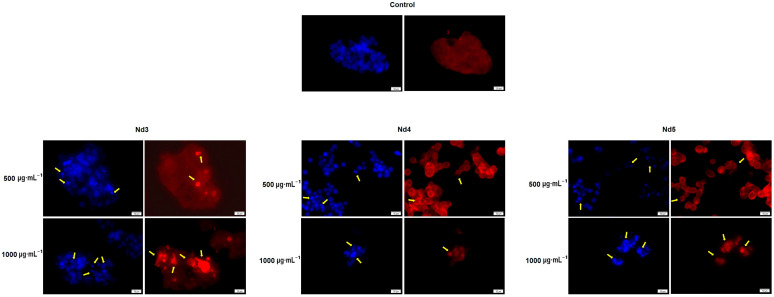
Aspect of the nuclei and F-actin fibers in MCF-7 cells treated for 48 h with Nd3, Nd4, and Nd5 at two representative concentrations (500 and 1000 μg·mL^−1^). The scale bars indicate 50 µm.

**Figure 16:**
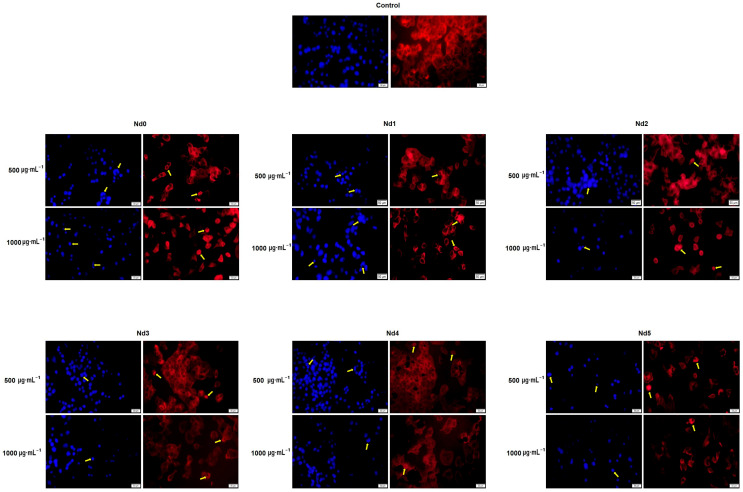
Aspect of the nuclei and F-actin fibers in PANC-1 cells treated for 48 h with Nd0, Nd1, Nd2, Nd3, Nd4, and Nd5 at two representative concentrations (500 and 1000 μg·mL^−1^). The scale bars indicate 50 µm.

Figure 14, Figure 15 and Figure 16 illustrate the changes in nuclear morphology and F-actin organization in A375, MCF-7, and PANC-1 cancer cells after 48 h treatment with Nd0-Nd5 at two representative concentrations: 500 and 1000 μg·mL^−1^. In all cell lines, control cells displayed a normal nuclear and cytoskeletal morphology; the nuclei presented an oval or sphere-like shape with evenly distributed chromatin that lacked signs of condensation, alongside well-organized F-actin fibers that were distributed throughout the cytoplasm without evidence of constriction.

In contrast, treatment with Nd compounds induced noticeable changes in their morphology, including nuclear condensation and fragmentation, as well as constriction of F-actin bundles, especially at the periphery of the cells. Additional signs of cytotoxicity, such as cell rounding and shrinkage, were also observed (Figure 14, Figure 15 and Figure 16).

Overall, the physicochemical investigations confirmed an effective incorporation of Nd into the cobalt ferrite structure up to z = 0.02 without secondary phase formation. Higher Nd concentrations resulted in the synthesis of orthorhombic NdFeO_3_. Increasing Nd content resulted in a gradual decline in particle size, as well as a drop in saturation and remanent magnetization. Biological testing indicated a selective, dose-dependent antiproliferative effect on the A375, MCF-7, and PANC-1 cancer cell lines, with little to no cytotoxic effects recorded on normal HaCaT cells at low to moderate concentrations. The potency of the cytotoxic effect was related to Nd content, as evidenced by LDH release tests and morphological apoptosis.

## 4. Discussion

Metal nanoparticles are a hot topic in anticancer research, a pathology that continues to raise worldwide challenges in terms of morbidity and mortality. Among such nanoparticles, cobalt ferrites triggered numerous studies due to their potential as both imagistic and therapeutic agents. The current study aimed to investigate the biological activity of newly synthesized Nd-doped cobalt ferrites, which should hypothetically benefit from the anticancer properties of all components. Cobalt ferrites have already been proven to be promising, highly selective anticancer agents that simultaneously display the ability to act as a platform for deciphering and controlling magnetic properties through structural chemistry manipulation [18].

Additionally, Nd can engage with organic molecules, resulting in complexes that are stable and have a high biocompatibility; its complex with phenantroline exhibited strong and selective cytotoxic effects [19]. Similarly, Nd formed a stable complex with tungstogermanate and 5-fluorouracil, which exhibited significant cytotoxic activity against two cancer cell lines, where pure 5-fluorouracil induced lower anticancer effects [20].

We aimed to synthesize Nd-doped cobalt ferrite nanoparticles that were further physicochemically analyzed by employing specific procedures.

The XRD analysis shows the successful incorporation of Nd within the cubic network. The combustion method used in this case for the synthesis of doped and undoped cobalt ferrite ensured optimal conditions for the development of single-phase cubic CoFe_2-z_Nd_z_O_4_ (Fd3m group) in samples Nd0, Nd1, and Nd2 (z = 0; 0.01 and 0.02, respectively). The generation of a secondary orthorhombic phase, NdFeO_3_ (Pbnm group), was reported in samples Nd3–Nd5 (z = 0.03; 0.05; 0.1, respectively). Furthermore, in sample Nd3 (z = 0.03), only a small peak was noticed, suggesting a trace amount of the secondary phase, which did not alter the characteristic patterns of the Nd-doped cobalt ferrite in terms of position and intensity. Muskan et al. [21] also reported the formation of the orthoferrite NdFeO_3_ as a secondary phase in their study that focused on CoNd_x_Fe_2-x_O_4_ (x = 0.0–0.6) nanoparticles.

Our results are consistent with those reported by Wang et al. [22], who used nitrates, citric acid, and the sol–gel self-propagating method to synthesize CoNd_x_Fe_2-x_O_4_ (0 ≤ x ≤0.2) nanoparticles. They showed that a doping amount of Nd that exceeded x = 0.05 led to the formation of NdFeO_3_ secondary phase in addition to the cubic cobalt ferrite. In another study focused on Nd-doped cobalt ferrite, Muskan et al. [23] identified x = 0.05 as the molar ratio that resulted in the formation of a secondary phase. In the current study, we synthesized CoFe_2-z_Nd_z_O_4_ nanoparticles along with NdFeO_3_ detected as a secondary phase; this was confirmed for z = 0.05, while only traces were present for z = 0.03. The presence of the secondary phase can be explained by the difference in the ionic radius between elements, the larger ionic radius (1.07 Å) of Nd substituting the smaller ionic radius (0.64 Å) of iron within the crystal lattice. Therefore, only a limited amount of Nd will be accommodated in the spinel structure.

The lattice parameter *a* from the cubic spinel network increased with the increase in the Nd content, in all tested samples, as revealed by the Rietveld refinement. This suggests that a higher amount of Nd successfully entered the cubic lattice. Consequently, the presence of Nd with a higher atomic radius caused the expansion of the unit cell, and simultaneously of the lattice parameter. The EDAX analysis showed that the characteristic band for Nd is present in samples Nd1–Nd5, which, combined with the increase in lattice parameter, can be interpreted as proof for the successful inclusion of Nd into the spinel lattice.

The FTIR spectra of both doped and undoped samples show similar absorption bands, characteristic of cobalt ferrite. The intense band located at approximately 570 cm^−1^ may be due to the metal–oxygen stretching vibration of the tetrahedral sites, while the partially visible band, at around 400 cm^−1^, can be attributed to the metal-oxygen stretching vibration from the octahedral sites. For samples Nd4 and Nd5, a small band was noticed around 3500 cm^−1^ that can be allocated to the -OH stretching vibration [24]. In addition, a small shift in the absorption band occurred, from 572 cm^−1^ in Nd0 to 568 cm^−1^ in Nd5, which contains the highest dopant quantity. This small shift can be attributed to the presence of Nd, whose amount triggers the band movement towards lower wavenumbers due to alterations in cation distribution, as well as lattice distortion. Furthermore, the band is attributed to the metal–oxygen stretching vibration from tetrahedral sites, so the shift could also indicate a tampering in the metal–oxygen bond [25]. Moreover, Iram et al. [26] studied lanthanum- and Nd-doped cobalt–strontium ferrite and underlined the build-up tension in the spinel structure caused by the presence of higher ionic radius rare earth elements. Additionally, they mentioned that during cation redistribution at the octahedral site, the ions of the doping elements could overlap the iron ions, leading to distortions at the interstitial positions.

Wu et al. [27] synthesized CoFe_1.9_RE_0.1_O_4_ (RE = Ho^3+^, Sm^3+^, Tb^3+^, Pr^3+^) nanoparticles by means of the hydrothermal method and similarly reported the two bands characteristic for cobalt ferrite, as well as a slight shift in the absorption maximum for the doped samples. Moreover, due to the affinity of rare earth elements for octahedral sites, as well as the formation of vacancies, cation redistribution, and a potential migration of iron from octahedral to tetrahedral sites, the metal–oxygen vibration will be altered, indicating changes in the Fe-O bond length and strength [22,28].

The magnetic measurements revealed a linear decrease in both mass magnetization Ms and remnant magnetization Mr with the increase in the Nd content. While for the undoped sample Nd0, the values of Ms and Mr are 76.55 emu∙g^−1^ and 33.58 emu∙g^−1^, respectively, in sample Nd5, the values decrease to 64.97 emu∙g^−1^ and 28.38 emu∙g^−1^, respectively. The reduced magnetization was also reported in another CoFe_2-x_Nd_x_O_4_ study [23], where the Ms starting value of 54.61 emu∙g^−1^ for the undoped sample dropped to 34.53 emu∙g^−1^ for x = 0.06. Furthermore, Wang et al. [22] noted a similar impact exerted by Nd on the magnetic properties of the resulting nanoparticles, stating that the phenomenon could be considered an indirect proof that Nd has successfully entered the ferrite lattice.

The magnetization reduction can be explained by the substitution of the smaller ionic radius Fe^3+^ with the larger ionic radius Nd^3+^. As previously mentioned, this notable difference in ionic radius disrupts the crystal structure, leading to lattice distortion, cation redistribution between sites, and the occurrence of defects. Moreover, the replacement of Fe^3+^ with Nd^3+^ in the B site will impair the super exchange interaction between A (tetrahedral) and B (octahedral) sites; according to Wang et al. [22], the super exchange interaction between A and B sites (Fe^3+^_A_^—^O^2—^Fe^3+^_B_ or Fe^3+^_A_^—^O^2—^Co^2+^_B_) are weakened, while the interactions from B and B sites (Fe^3+^_B_^—^O^2—^Co^2+^_B_ or Fe^3+^_B_^—^O^2—^Fe^3+^_B_) will exhibit an increase in the negative exchange, showing stronger antiferromagnetic behavior.

In addition, a contribution to the decline of the magnetic properties can also be attributed to the presence of the NdFeO_3_ secondary phase that could disrupt the interaction within the crystal lattice [29].

The STEM analysis revealed that the reaction yielded particles with dimensions between 10 and 46 nm. The insertion of Nd into the ferrite lattice caused a decrease in particle size depending on the Nd content; thus, the smallest nanoparticles (10–30 nm) were identified in sample Nd5, which contained the highest amount of the doping element. In our previous study [30], we focused on dysprosium-doped cobalt ferrite CoFe_2-x_Dy_x_O_4_ (x = 0; 0.1; 0.2; 0.4) and achieved comparable results, with increasingly reduced nanoparticle size as the dysprosium content increased. Similar findings were described by Yusafi et al. [28], who synthesized CoNd_x_Fe_2-x_O_4_ for x = 0.0–0.5 and recorded a particle decrease with the increase in the Nd content. They also noted an intense particle agglomeration as well as a tendency towards irregular nanoparticle shape following Nd insertion into the ferrite lattice. As previously explained, this phenomenon is caused by the difference in ionic radius between iron and Nd, which induces alterations in the spinel lattice. Furthermore, Wang [22] noted that, in addition to the Nd insertion in the crystal lattice, the emergence of the secondary phase can also alter the normal formation of the ferrite grains. As an example, in gadolinium-doped nickel ferrites [31], the particle size decrease was justified by the pressure created by Gd^3+^ ions at the grain boundaries, leading to growth restriction. Moreover, in the case of praseodymium-, holmium-, terbium-, and samarium-doped cobalt ferrite, Wu et al. [27] observed that all rare earths induced a particle size reduction, attributed to the localization of the dopants at the boundaries of the grain, thereby exerting pressure and inhibiting their growth.

The newly synthesized nanoparticles were tested in terms of antiproliferative activity against three cancer cell lines, aiming to identify a potential correlation between the Nd content and the respective biological effect. The antiproliferative activity was assessed using the Alamar Blue assay. The antiproliferative effect manifested in a dose-dependent manner; the lowest cell viability was reported for the highest applied concentrations of the tested samples. Remarkably, the Nd content significantly influenced the cell viability-decreasing action of the respective compound, its increase being directly proportional to the potency of the antiproliferative action. As clearly shown by previous studies, cobalt ferrites induce anticancer effects observed in multiple cancer cells, a fact validated in our study by the behavior of the Nd0 sample; however, the insertion of Nd into the cobalt ferrite crystal lattice increased the sample’s antiproliferative effects in all three cancer cell lines. The cytotoxic effect exerted by Nd in MCF-7 cancer cells was revealed by several studies that investigated Nd complexes [32], Nd_2_O_3_ nanoparticles [33], Nd-doped carbon dots [34], and Nd^3+^-doped GdPO_4_ core nanoparticles [35]; in all studies, the cytotoxic activity was accompanied by reduced toxicity. The gradual increase in Nd content from sample Nd1 to Nd5 is accompanied by a proportional decrease in the nanoparticle’s size, which could explain the increased antiproliferative activity by the increased surface-to-volume ratio that ensures a better contact between the nanoparticle and the biological environment. Most importantly, the presence of Nd is essential for the antiproliferative activity; in the form of oxide, Nd_2_O_3_, it triggers the production of ROS able to alter, in a dose-dependent fashion, the function of cellular components such as DNA, protein, and lipids and induce cell death [36]. Such effects add to the anticancer properties of the cobalt ferrite itself, which significantly decreased MCF-7 cell viability following uptake or adsorption by means of apoptosis induction [37]. When screening for anticancer therapeutic options, one must investigate the selectivity of newly developed agents in order to avoid systemic toxicity; one suitable test is in vitro cell viability in normal cells. We used HaCaT cells (immortalized human keratinocytes) as the non-malignant control, given their stable phenotype, reproducibility, and wide use as research material in cytotoxicity studies [38]. It is of note that HaCaT cells showed no loss of viability at concentrations up to 500 μg·mL^−1^; moreover, some samples (e.g., Nd1, Nd2) even increased cellular viability, as observed in the Alamar Blue assay. Only when the sample concentration increased to 1000 μg·mL^−1,^ HaCaT cell viability decrease. In contrast, cancer cell lines displayed a significant reduction in viability at 500 μg·mL^−1^, with PANC-1 in particular showing a marked sensitivity at lower concentrations, in both Alamar Blue and LDH assays. LDH release measurements confirmed these findings, indicating minimal membrane damage in HaCaT cells at concentrations lower than 500 μg·mL^−1^, whereas cancer cells, especially PANC-1, exhibited a dose-dependent cytotoxicity starting at lower concentrations. Overall, these results indicate a potential therapeutic application below the maximal tested dose (1000 μg·mL^−1^) and support a preferential cytotoxic effect toward malignant cells at 500 µg·mL^−1^; such behavior is characterized as a selective anticancer activity [16] which might hold promise for future therapeutic opportunities. Nonetheless, because HaCaT is not a tissue-matched cell line to the breast or pancreatic cancer cell line, we acknowledge that this is a limitation, and future studies will incorporate normal breast and pancreatic epithelial cell lines to more accurately assess tissue-specific selectivity.

In agreement with cell viability tests, the morphological assessment using an immunofluorescence assay revealed spherical nuclei with evenly distributed chromatin in control cells, accompanied by well-organized actin fibers within the cytoplasm. Treatment with metallic nanoparticles induced an altered morphology in all cancer cell types with nuclear condensation and fragmentation, and the constriction of the actin fibers. Cell shrinkage was also noticed, thus indicating apoptotic effects [39] whose intensity increased with the Nd content. Indeed, Nd was previously reported as an apoptosis inducer in mouse liver cells, but in its most water-soluble form as Nd nitrate, which was studied as an environmental pollutant [40]. When tested as Nd_2_O_3_ nanoparticles in non-small lung cancer cells, Nd exerted cytotoxic effects mainly through autophagy instead of apoptosis [41]. However, the apoptotic mechanism of cell death was previously reported for undoped cobalt ferrite nanoparticles in liver and colon cancer cells in a concentration-dependent manner [42]. To our knowledge, this is the first study suggesting that Nd-doped cobalt ferrite nanoparticles exert cytotoxic effects that may occur via apoptotic mechanisms, although further confirmatory studies are required to validate this proposed pathway.

In order to confirm their cytotoxic activity, we also assessed the release of lactate dehydrogenase in the tested cancer cells following cell membrane alterations at 48 h post-treatment. In all three cancer cells, the Nd-free sample Nd0 showed cytotoxic effects solely at high concentrations, while the Nd-doped samples significantly increased the LDH release. LDH is an enzyme that catalyzes the metabolic conversion of pyruvate to lactate in the cytosol, whose release in the extracellular space occurs following cell membrane damage or cell death, thus making LDH release a reliable marker of cytotoxic effects [43]. Cobalt ferrite nanoparticles were already reported to induce increased LDH release in A549 lung cancer cells [44]. Our results confirm such results, which also validate cell viability tests.

While this study demonstrates that Nd-doped cobalt ferrite nanoparticles affect cancer and normal cells differently, the mechanisms underlying this selectivity remain to be fully elucidated. Given that cancer cells often exhibit altered oxidative metabolism, including elevated ROS production and a more acidic intracellular environment [45,46], future investigations should explore how these factors interact with nanoparticle-induced ROS production. These pH-dependent effects, combined with the intrinsic differences in redox homeostasis between cancer and normal cells, could contribute to the selective cytotoxicity observed [47,48]. Studies examining the nanoparticles’ impact on ROS levels, oxidative enzyme activity, and the expression of genes regulating oxidative metabolism would provide valuable insight into their mode of action.

## 5. Conclusions

The present research focuses on the design and synthesis of Nd-doped cobalt ferrite nanoparticles by means of a straightforward combustion method, followed by their physicochemical analysis. Although Nd-doped metallic nanoparticles have been reported before, as per our knowledge goes, this is the first study that reports the biological effects of Nd-doped cobalt ferrites whose design aimed to unite the anticancer benefits of both the rare earth element and the cobalt ferrite. Five types of doped nanoparticles were synthesized using increasing amounts of Nd (Nd1–5), while undoped cobalt ferrite nanoparticles (Nd0) were used as a reference. Samples were assessed in terms of shape, size, morphology, and magnetic properties; a clear correlation occurred between size and Nd content, whose increase triggered a diameter decrease. Cell viability assay revealed significant antiproliferative effects for both undoped and doped cobalt ferrite nanoparticles; the presence of Nd in the crystal lattice was correlated with increased reduction in cell viability compared to the undoped sample. Moreover, results showed that a relationship of direct proportionality could be established between the amount of the doping element and the potency of cell viability reduction. Using conventional cytotoxic tests such as morphological assay and lactate dehydrogenase release, the findings suggest that apoptosis may be involved as a potential mechanism of cell death. Further studies are necessary to deliver more comprehensive information on the exact mechanism of action at the cellular level and their potential as anticancer agents.

## Figures and Tables

**Table 1:** The sample composition.

Sample	NdCl_3_·6H_2_O (Moles)	Fe(NO_3_)_3_·9H_2_O (Moles)
Nd0	-	0.0400
Nd1	0.0002	0.0398
Nd2	0.0004	0.0396
Nd3	0.0006	0.0394
Nd4	0.0010	0.0390
Nd5	0.0020	0.0380

**Table 2:** Calculated lattice parameter *a*.

Sample	Lattice Parameter *a* (Å)
Nd0	8.3762 ± 0.0003
Nd1	8.3781 ± 0.0004
Nd2	8.3796 ± 0.0004
Nd3	8.3799 ± 0.0004
Nd4	8.3830 ± 0.0004
Nd5	8.3873 ± 0.0002

**Table 3:** The calculated IC_50_ values (µg·mL^−1^) of Nd0, Nd1, Nd2, Nd3, Nd4 and Nd5 in HaCaT, A375, MCF-7 and PANC-1 cells after 48 h treatment.

	HaCaT	A375	MCF-7	PANC-1
Nd0	>1000	>1000	>1000	748.7
Nd1	>1000	>1000	>1000	687.9
Nd2	>1000	>1000	>1000	593.5
Nd3	>1000	943.5	>1000	581.8
Nd4	>1000	798.8	956.3	572.3
Nd5	>1000	650.7	877.2	397.7

## Data Availability

The original contributions presented in the study are included in the article, further inquiries can be directed to the corresponding author.
